# Removal of Cr(VI) from Aqueous Solutions Using Powder of Potato Peelings as a Low Cost Sorbent

**DOI:** 10.1155/2014/973153

**Published:** 2014-06-29

**Authors:** Farai Mutongo, Olga Kuipa, Pardon K. Kuipa

**Affiliations:** ^1^Department of Chemical Engineering, National University of Science and Technology, Bulawayo, Zimbabwe; ^2^School of Engineering Sciences and Technology, Chinhoyi University of Technology, Private Bag Box 7724, Chinhoyi, Zimbabwe

## Abstract

Potato peels which are a low cost, renewable agroindustry by-product were used for the removal of hexavalent chromium from aqueous effluents. Batch experiments were carried out with an artificial effluent comprising of potassium dichromate in deionised water. The effects of the initial hexavalent chromium concentration, dose of biosorbent, and removal kinetics were explored. An adsorbent dosage of 4 g/L was effective in complete removal of the metal ion, at pH 2.5, in 48 minutes. The kinetic process of Cr(VI) adsorption onto potato peel powder was tested by applying pseudo-first-order and pseudo-second-order models as well as the Elovich kinetic equation to correlate the experimental data and to determine the kinetic parameters. The adsorption data were correlated by the Langmuir and Freundlich isotherms. A maximum monolayer adsorption capacity of 3.28 mg/g was calculated using the Langmuir adsorption isotherm, suggesting a functional group limited adsorption process. The results confirmed that potato peels are an effective biosorbent for the removal of hexavalent chromium from effluent.

## 1. Introduction

Wastewater from industries such as chrome leather tanning, metallurgy, chrome plating, textiles, ceramics, photography, and photoengraving contains moderate to excessive amounts of hexavalent chromium compounds beyond the conventional statutory limit of 0.1 mg/L. Remediation of these effluents is necessary because in humans Cr(VI) causes lung cancer, ulcers, nasal septum perforations, and damage to the kidneys.

Established methods for the removal of chromium from wastewaters include precipitation, electrochemical reduction, ion exchange, electrodeposition, membrane technology, and adsorption. Adsorption remains one of the most economical and widely used method for the removal of toxic pollutants from wastewater and the most widely used Cr(VI) sorbent is activated carbon although it is expensive and has high running costs since it requires regeneration after sorption. Therefore the potential exists for Cr(VI) removal by a lower cost biosorbent. The use of nonliving biomass as metal binding compounds has gained popularity over the use of live biomass. This is mainly because living cells are subject to toxic effects of heavy metals, resulting in cell death. Living cells also require the addition of nutrients and hence increase the BOD and COD in the effluent. Dead cells on the other hand require little care and maintenance, are cheaper, and can be easily regenerated and reused.

The adsorption of Cr(VI) by a number of materials such as agroindustry waste residues [1–4], forestry waste [5, 6], fruit peelings and leaves [7–10], fungi [11, 12], dead bacterial, and diatom biomass [13–15] has been reported in the literature.

This study investigated the use of potato peels which are widely available in many countries as a biosorbent for a low cost Cr(VI) removal technology. The Langmuir and Freundlich isotherms were employed to analyse the equilibrium data. The Elovich, pseudo-first-order, and pseudo-second-order kinetic models were used for the kinetic interpretation of the adsorption data. The effects of varying concentration of the chromium, contact time, and adsorbent dose were investigated.

## 2. Materials and Methods

### 2.1. Preparation of Chromium Solution and Analysis

A stock solution (500 mg/L) was prepared by dissolving 1.4144 g of AR grade potassium dichromate in one liter of deionised water. Experimental solutions of the desired concentrations were obtained from the stock solution by appropriate dilutions with deionised water. Cr(VI) was quantified using diphenylcarbazide (DPC) which forms a red violet complex whose intensity was read at 540 nm using a Hach Spectrophotometer (model DR 2010) as outlined in the* Standard Methods for the Examination of Water and Wastewater* handbook [16]. All glassware was cleaned with 6 mol/L HCl and then rinsed in deionised water.

### 2.2. Preparation of Biosorbent

Potato peels, referred to as biosorbent, were collected from a local food chain (Chicken Inn) free of charge and washed in deionized water and then rinsed in 0.1 M HCl. In acidic media the surface of the sorbent is expected to be protonated to a large extent resulting in a stronger attraction for chromium oxyanions in the solution (in acidic media Cr(VI) exists in the form of oxyanions such as HCrO_4_^−^, CrO_4_^−^, Cr_2_O_7_^2−^, Cr_3_O_10_^2−^). The biosorbent was left to dry at approximately 103–110°C overnight in an oven. The dried biosorbent was then ground to 100% passing 75 *μ*m and stored in airtight plastic bottles prior to batch tests.

## 3. Batch Biosorption Studies

For all the batch biosorption studies blank experimental runs with only the adsorbent in 100 mL of distilled water were conducted simultaneously at similar conditions to account for any adsorbate leached by the adsorbents and adsorbed by the glass container walls. All batch experiments were carried out at pH 2.5 and all mixtures were stirred by a magnetic stirrer at 300 rpm. The value of the pH was chosen based on previous research that reported that the point of zero charge for potato peel biomass is at pH 6.59 [17], meaning that the biomass has an overall positive charge for pH values below 6.59 and starts to acquire an overall negative charge for pH values above 6.59. At pH values that are too low it is expected that hydronium ions, H_3_O^+^, would associate with the adsorbent surface sites thus restricting access to the surface sites by the metal ions through repulsive forces. A pH value of 2.5 was chosen to avoid metal ion precipitation. All experiments were conducted at ambient temperature (27°C).

### 3.1. Effect of Absorbent Dose

Absorbent dose, varied from 0.5 to 4.0 g/L, was added to 100 mL of 40 mg/L K_2_Cr_2_O_7_ in 100 mL volumetric flasks. The pH was adjusted to 2.5 using 0.1 M HCl. Approximately 10 mL of solution for analysis was withdrawn from each flask at 24-minute intervals and filtered. The filtrate from each flask was then analyzed for residual Cr(VI) concentration.

### 3.2. Effect of Initial Cr(VI) Concentration with Time

The initial concentration of the potassium dichromate solution was varied from 20 to 120 mg/L. A constant absorbent dose of 4 g/L was used for this set of experiments. The pH was adjusted to 2.5 using 0.1 M HCl. The equilibrium curve was plotted from Cr(VI) percentage removal obtained after a time period of 148 minutes.

## 4. Results and Discussion

### 4.1. Effect of Absorbent Dose and Contact Time

**Figure 1:**
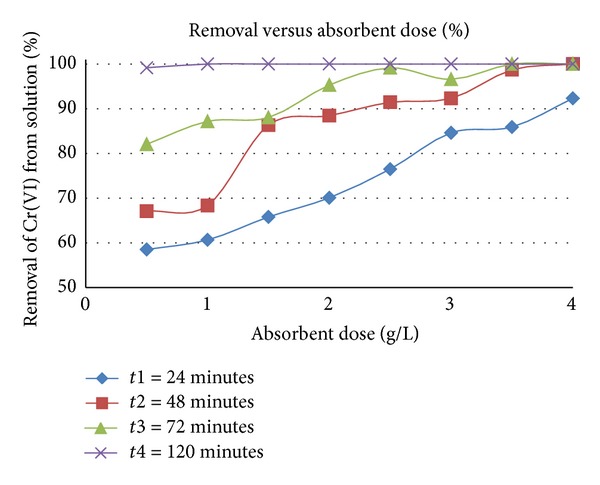
Effect of absorbent dose on % Cr(VI) removal as a function of time, pH 2.5, and initial Cr(VI) concentration of 40 mg/L.

**Figure 2:**
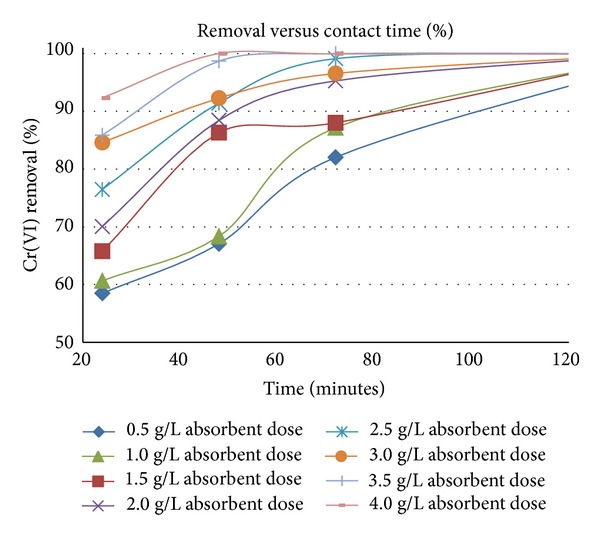
Effect of contact time on % Cr(VI) removal, pH 2.5, and initial Cr(VI) concentration of 40 mg/L.

Percentage removal of Cr(VI) from solution increased steadily with increase in absorbent dose and contact time (Figures 1 and 2). Complete removal is attained after only 48 minutes with a dosage of 4 g/L, whilst a dosage of 0.5 g/L requires 120 minutes to attain 96 ± 2% removal.

### 4.2. Effect of Initial Cr(VI) Concentration on Percent Removal

**Figure 3:**
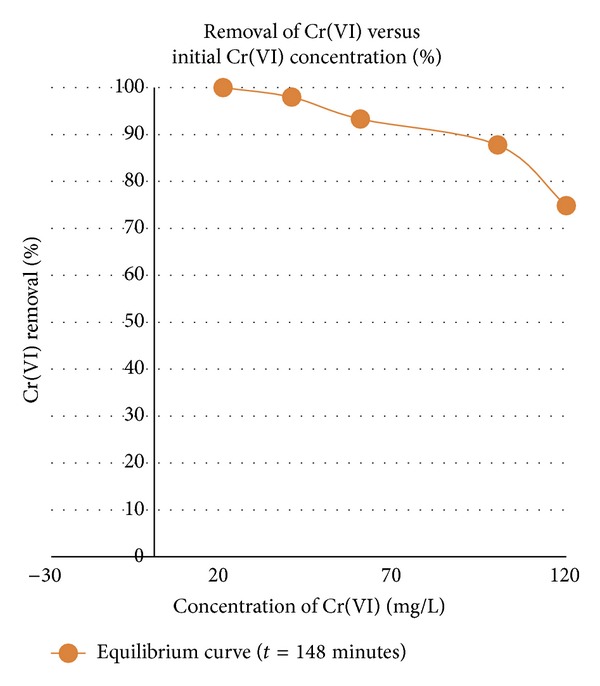
Effect of initial Cr(VI) concentration, pH 2.5, and absorbent dose of 4 g/L.

Cr(VI) sorption was studied in batch experiments (pH 2.5) using different initial Cr(VI) concentrations of 20, 40, 60, 100, and 120 mg/L (Figure 3).

Complete removal of Cr(VI) was attained for solutions up to 40 mg/L. The “final equilibrium curve,” was plotted from percentage removal obtained after a time period of 148 minutes. The equilibrium curve shows that the overall percent removal of Cr(VI) from solution decreases with an increase in initial Cr(VI) concentration. This may be attributed to lack of sufficient surface area to accommodate much more metal available in the solution. It is evident that the amount of chromium removed from solution increases with an increase in concentration of Cr(VI). This is probably due to the higher interaction between the metal ions and metal sequestering sites of the biosorbent. The final percentage removal for an initial chromium concentration of 120 mg/L was 74.84% whilst initial concentration of 100 and 60 mg/L saw a removal of 87.79% and 93.31%, respectively.

## 5. Adsorption Isotherm Investigation

Cr(VI) uptake was calculated from mass balance, the difference between initial and final chromium concentrations:

(1) $$\begin{array}{c} q\;\left(\text{mg}\;{\text{g}}^{-1}\right)=\frac{\left[C_{0}-C\right]\left(\text{mg}/\text{L}\right)}{m\left(\text{g}\right)}V\left(\text{L}\right), \end{array}$$

where *q* is the metal uptake; *C*_0_ and *C* are the initial and final Cr(VI) concentration. *m* is the mass of biosorbent and *V* is the volume of solution used.

### 5.1. Langmuir Isotherm

The Langmuir equation refers to a monolayer sorption onto surfaces containing a finite number of accessible sites:

(2) $$\begin{array}{c} q_{e}=\frac{q_{m}bC_{e}}{1+bC_{e}}. \end{array}$$

*b* and *q*_*m*_ are constants related to the apparent energy of sorption and the sorption capacity, respectively. *q*_*e*_ is the amount absorbed per unit mass of the absorbent (mg g^−1^) with an equilibrium concentration of *C*_*e*_ (mg L^−1^). The values of *b* and *q*_*m*_ were calculated from the slope and intercept of the linear plot of *C*_*e*_/*q*_*e*_ versus *C*_*e*_:

(3) $$\begin{array}{c} \frac{C_{e}}{q_{e}}=\frac{1}{bq_{m}}+\frac{C_{e}}{q_{m}}. \end{array}$$

The regression equation obtained is

(4) $$\begin{array}{c} y=0.305x+0.680,\\ R^{2}=0.973,\\ \text{the}\;\text{constants}\;q_{m}=3.28\;\text{mg}/\text{g},\;b=0.448\;\text{L}/\text{mg}. \end{array}$$

The essential feature of the Langmuir isotherm model can be expressed by means of a separation factor or equilibrium parameter (*R*_*L*_) which is calculated as

(5) $$\begin{array}{c} R_{L}=\frac{1}{1+bC_{0}}. \end{array}$$

The value of *R*_*L*_ indicates the type of biosorption isotherm to be linear (*R*_*L*_ = 1), favourable (0 < *R*_*L*_ < 1), unfavourable (*R*_*L*_ > 1), and irreversible (*R*_*L*_ = 0).

It can be noted that for this work, *R*_*L*_ *is favourable *since both *b* and *C*_0_ are positive values, which indicates favourable biosorption of chromium by potato peel powder.

### 5.2. Freundlich Isotherm

The Freundlich isotherm model was applied to study the biosorption behavior assuming a heterogeneous adsorption surface and active sites with different energy and its linearized equation is

(6) $$\begin{array}{c} \log q_{e}=\log K_{f}+n_{f}\log C_{e}. \end{array}$$

The Freundlich constants *k*_*f*_ and *n*_*f*_ are related to adsorption capacity and intensity, respectively, and were calculated from the slopes and intercept of the linear plot of log⁡⁡*q*_*e*_ versus log⁡⁡*C*_*e*_.

The linear regression equation obtained is given below with the calculated constants:

(7) $$\begin{array}{c} y=0.667x-0.525,\\ R^{2}=0.974,\\ n_{f}=0.667,\;\;K_{f}=0.2985. \end{array}$$

*n*_*f*_ lies between 0 and 1 indicating also favourable biosorption.

Both the Langmuir and the Freundlich adsorption models had a good fit to the equilibrium data which suggests that both monolayer and heterogeneous surface adsorption affect the biosorption.

## 6. Kinetic Modelling

### 6.1. Pseudo-First-Order Equation

**Figure 4:**
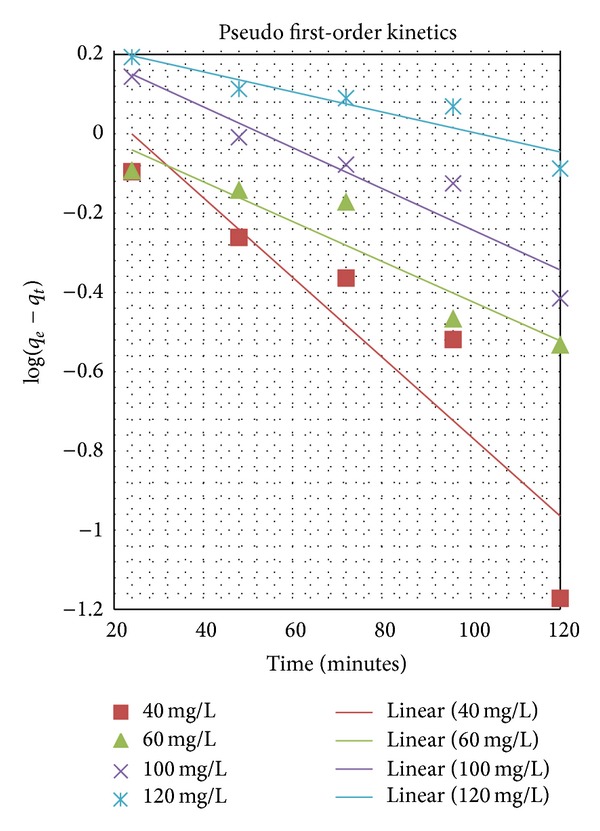
Pseudo-first-order kinetics, pH 2.5, ambient temperature, and 4 g/L absorbent dose.

Consider

(8) $$\begin{array}{c} \frac{dq_{t}}{dt}=k_{1}\left(q_{e}-q_{t}\right) \end{array}$$

which on integration and simplification becomes

(9) $$\begin{array}{c} \log(q_{e}-q_{t})=\log q_{e}-\frac{k_{1}}{2.303}t, \end{array}$$

where *q*_*t*_ (mg/g) is the metal uptake at time *t*, (min.), *q*_*e*_ is the metal uptake at equilibrium, and *k*_1_ is the pseudo-first-order kinetic model constant (min.). The values of *k*_1_ and *q*_*e*_ can be obtained from the slope and intercept of the plot of log⁡⁡(*q*_*e*_ − *q*_*t*_) versus *t*, respectively (Figure 4). The results are summarised in Tables 1, 2(a), and 2(b).

### 6.2. Pseudo-Second-Order Equation

Consider

(10) $$\begin{array}{c} \frac{{dq}_{t}}{dt}=k_{2}{\left(q_{e}-q_{t}\right)}^{2}. \end{array}$$

And the linear form is

(11) $$\begin{array}{c} \frac{t}{q_{t}}=\frac{1}{k_{2}q_{e}^{2}}+\frac{1}{q_{e}}\cdot t, \end{array}$$

where *k*_2_ is the pseudo-second-order kinetic constant (g mg^−1 ^min^−1^). *k*_2_ and *q*_*e*_ can be calculated from the slope and intercept of the plot of *t*/*q*_*t*_ versus *t*. The results are summarised in Table 1. Low correlation coefficients for the pseudo-second-order kinetics model suggest that the model is not applicable to Cr(VI) adsorption onto potato peel powder, implying that the rate limiting step is not chemical adsorption.

### 6.3. Elovich Kinetic Equation

The Elovich kinetic equation is for general application to chemisorptions kinetics. It suggests that the active sites are heterogeneous in nature and therefore exhibit different activation energies for chemisorption. The equation is based on the adsorption capacity of the sorbent:

(12) $$\begin{array}{c} \frac{{dq}_{t}}{dt}=\alpha \cdot \exp\left(-\beta q_{t}\right), \end{array}$$

where *q*_*t*_((g/kg)/min⁡⁡) and *q*_*e*_ is the metal uptake at equilibrium (mg/g), *α* is the initial adsorption rate (mg*·*g^−1 ^min^−1^), and *β* is the desorption constant (g*·*mg^−1^).

Simplification is attained by making the assumption, *αβ* ≫ *t* [18, 19].

With boundary conditions *q*_*t*_ = 0, *t* = 0, *q*_*t*_ = *q*_*t*_, and *t* = *t*,

(13) $$\begin{array}{c} q_{t}=\frac{1}{\beta }\ln\left(\alpha \beta \right)+\frac{1}{\beta }\ln t. \end{array}$$

**Figure 5:**
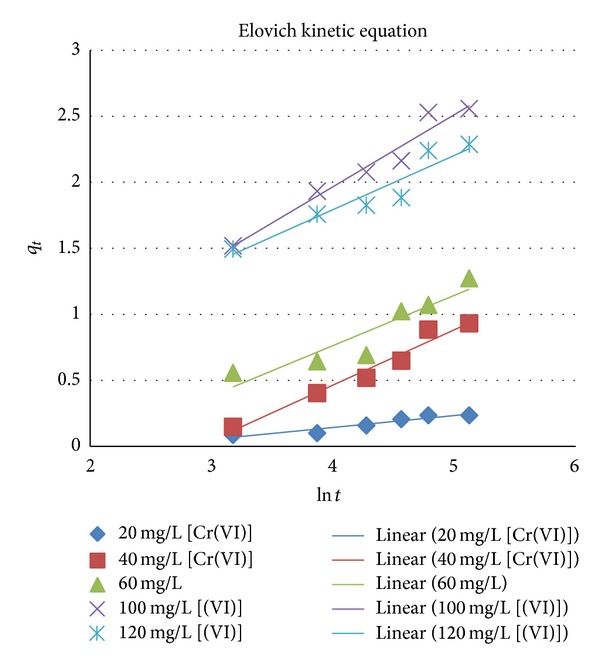
The Elovich kinetic model, pH 2.5, ambient temperature, and absorbent dose of 4 g/L.

The constants can be calculated from the slope and intercept of the plot of *q*_*t*_ versus ln *t* (Figure 5). The results are summarised in Tables 1, 2(a), and 2(b).

## 7. Comparison of the Present Study with Literature

The data in Table 3 shows that there is a wide variation in the maximum absorption capacity of Cr(VI) by biosorbents (based on the Langmuir isotherm). The maximum adsorption capacity in this study is lower than that reported elsewhere in the literature [4].

## 8. Conclusions

When applying the Langmuir isotherm to the adsorption data, a maximum monolayer adsorption capacity of 3.28 mg/g suggests a functional group limited adsorption process. Indeed potato peel has been reported [23] to be a source of phenolic compounds, glycoalkaloids, and cell wall polysaccharides. These compounds would be the source of the functional groups. The value of the adsorption energy, *b*, was found to be 0.448 L/mg. When the adsorption data are modeled using the Freundlich isotherm, the equilibrium constant, *K*_*f*_, is found to be 0.2985 ((mg/g) (L/mg)^1/*n*^). The value of *n* was 2/3, which is between 0 and 10, suggesting relatively strong adsorption of Cr(VI) ions onto the surface of the potato peel powder. Low correlation coefficients for the pseudo-second-order kinetics model suggest that the model is not applicable to Cr(VI) adsorption onto potato peel powder, implying that the rate limiting step is not chemical adsorption. Relatively high correlation coefficients (*R* ^2^) for the pseudo-first-order model and the Elovich kinetic equation suggest that these models can adequately describe the kinetic process of Cr(VI) adsorption onto potato peel powder. Applicability of the Elovich model implies that the active sites of the potato peel powder are heterogeneous in nature and therefore exhibit different activation energies for chemisorption. In general, it may be concluded that the use of potato peel powder is an effective method for the abatement of Cr(VI) aqueous contaminants.

## Figures and Tables

**Table 1:** Regression equations and correlation factors using pseudo-first-order, pseudo-second-order, and Elovich kinetic models for the biosorption of Cr(VI) by waste potato peel.

[Cr(VI)] mg/L	Pseudo-first-order kinetics	Pseudo-second-order kinetics	Elovich kinetics
40	*y* = − 0.01*x* + 0.274 **R** ^2^ = **0.842**	*y* = − 0.569*x* + 223.9 *R* ^2^ = 0.153	*y* = 0.418*x* − 1.217 **R** ^2^ = **0.965**
60	*y* = − 0.005*x* + 0.080 **R** ^2^ = **0.882**	*y* = 0.146*x* + 94.60 *R* ^2^ = 0.122	*y* = 0.380*x* − 0.757 **R** ^2^ = **0.861**
100	*y* = − 0.005*x* + 0.274 **R** ^2^ = **0.906**	*y* = 0.114*x* + 36.64 *R* ^2^ = 0.139	*y* = 0.410*x* + 0.149 **R** ^2^ = **0.902**
120	*y* = − 0.002*x* + 0.257 **R** ^2^ = **0.870**	*y* = 0.084*x* + 46.70 *R* ^2^ = 0.048	*y* = 0.546*x* − 0.218 **R** ^2^ = **0.956**

**(a):** 

Kinetic model	Linear equation	Initial Cr(VI) concentration, mg/L	Reaction constant	*R* ^2^
Pseudo-first-order	$\log(q_{e}-q_{t})=\log q_{e}-\frac{k_{1}}{2.303}t$	40	*k*_1_ = 0.0230 min^−1^	0.842
		60	*k*_1_ = 0.0115 min^−1^	0.882
		100	*k*_1_ = 0.0115 min^−1^	0.906
		120	*k*_1_ = 0.0046 min^−1^	0.870

Elovich	$q_{t}=\frac{1}{\beta }\ln\left(\alpha \beta \right)+\frac{1}{\beta }\ln t$	20	*α* = 0.0081 mg*·*g^−1^ *·*min^−1^ *β* = 11.11 g/mg	0.907
		40	*α* = 0.0227 mg*·*g^−1^ *·*min^−1^ *β* = 2.3923 g/mg	0.965
		60	*α* = 0.0518 mg*·*g^−1^ *·*min^−1^ *β* = 2.6316 g/mg	0.861
		100	*α* = 0.5897 mg*·*g^−1^ *·*min^−1^ *β* = 2.4390 g/mg	0.902
		120	*α* = 0.3663 mg*·*g^−1^ *·*min^−1^ *β* = 1.8315 g/mg	0.956

**(b):** 

Kinetic model	Linear equation	Reaction constant
Pseudo-first-order	$\log(q_{e}-q_{t})=\log q_{e}-\frac{k_{1}}{2.303}t$	*k*_1_ = 0.01267 ± 0.00764 min^−1^
Elovich	$q_{t}=\frac{1}{\beta }\ln\left(\alpha \beta \right)+\frac{1}{\beta }\ln t$	*α* = 0.25763 ± 0.3440 mg*·*g^−1^ *·*min^−1^ *β* = 2.3236 ± 0.27054 g/mg

**Table 3:** Comparison between the results of this work and other results found in the literature for the monolayer maximum adsorption capacity of Cr(VI) ions with various adsorbents.

Biosorbent	*Q*_*m*_ (mg/g)	Reference
*Agave lechuguilla *	33.55	[18]
Brown seaweed	0.629	[20]
Maize husk	28.49	[2]
Maize bran	312.52	[3]
Potato peel waste	8.012	[4]
*Psidium guajava* leaves powder	4.762	[8]
Modified pomegranate peel	13.01	[9]
Formaldehyde modified pomegranate peel	22.28	[9]
Powder of mosambi fruit peel	7.51	[10]
*Acacia nilotica* leaf	69.4	[5]
Pine cone and oak cups	Ranges from 4.19 to 7.48	[6]
Pine leaves	0.198	[7]
Sawdust	0.470	[7]
Raw and modified palm branches	55.0	[21]
Dried biomass of cyanobacterium *Oscillatorialaetevirens *	103.09	[13]
Diatom *Planothidium lanceolatum *	93.45	[14]
Biomass of *Trichoderma gamsii *	44.8	[15]
Dead biomass of green algae *Spirogyra* spp.	265.0	[22]
Tea fungus	58.0	[11]
*Trametes versicolor Polyporus* fungi	125.0	[12]
Potato peel powder	3.28	Present Study
